# Prenatal-Onset Lethal Adenylosuccinate Lyase Deficiency Diagnosed by Rapid Whole Genome Sequencing: A Case Report

**DOI:** 10.7759/cureus.109493

**Published:** 2026-05-23

**Authors:** Jiri Dusek, Lenka Nedvedova, Eva Rysava, Josef Bartl, Petra Pokorna, Katerina Slaba

**Affiliations:** 1 Faculty of Health and Social Science, University of South Bohemia in České Budějovice, České Budějovice, CZE; 2 Department of Neonatology, Hospital České Budějovice, a.s., České Budějovice, CZE; 3 Department of Genetics, Hospital České Budějovice, a.s., České Budějovice, CZE; 4 Department of Pediatrics and Inherited Metabolic Disorders, General University Hospital in Prague, Prague, CZE; 5 Department of Biology, Centre for Precision Medicine, University Hospital, Faculty of Medicine, Masaryk University Brno, Brno, CZE; 6 Department of Pediatrics, Centre for Precision Medicine, University Hospital, Faculty of Medicine, Masaryk University Brno, Brno, CZE

**Keywords:** adenylosuccinate lyase deficiency, epileptic encephalopathy, microcephaly, neonatal, rapid whole genome sequencing, succinylpurines

## Abstract

Adenylosuccinate lyase deficiency (ADSLD) is an ultra-rare autosomal recessive disorder of purine metabolism. The most severe neonatal phenotype presents with profound encephalopathy from birth, hypotonia, respiratory insufficiency, and early seizures.

A preterm infant was delivered at 34+0 weeks with antenatal progressive microcephaly and mild polyhydramnios. At birth, the neonate exhibited absent spontaneous respiration and marked hypotonia, necessitating ventilation and intubation. Respiratory distress syndrome (RDS) was managed with surfactant, but two extubation attempts with non-invasive continuous positive airway pressure/biphasic positive airway pressure (nCPAP/DuoPAP) were unsuccessful. Neurologically, the infant demonstrated profound central hypotonia, stimulus-induced myoclonus, and a burst-suppression pattern on amplitude-integrated electroencephalography (aEEG). Antiseizure therapy with phenobarbital and levetiracetam was initiated. Brain magnetic resonance imaging (MRI) revealed microcephaly with simplified gyrification, bilateral intraventricular hemorrhage (IVH) with additional posterior fossa hemorrhage, delayed myelination, and evolving post-hemorrhagic hydrocephalus. Targeted biochemical testing identified markedly elevated succinylpurines (succinyladenosine (S-Ado) and SAICA-riboside (SAICAr)) in urine and serum. Rapid whole-genome sequencing (WGS) confirmed autosomal recessive ADSLD. In the absence of disease-modifying therapy and given the extremely poor prognosis, care was transitioned to comfort-focused palliation. ADSLD should be considered in neonates presenting with severe microcephaly, profound hypotonia, and early epileptic encephalopathy. Rapid genomic testing, when available and clinically appropriate, may enable timely etiologic diagnosis, supporting prognostication and shared decision-making.

## Introduction

Adenylosuccinate lyase deficiency (ADSLD) is an ultra-rare autosomal recessive inborn error of purine metabolism caused by pathogenic variants in the ADSL gene. Adenylosuccinate lyase (ADSL) catalyzes two steps in de novo purine synthesis, and its deficiency leads to the accumulation of succinylpurines, primarily succinyladenosine (S-Ado) and SAICA-riboside (SAICAr), which represent the biochemical signature of the disease and can be detected in urine, plasma, and cerebrospinal fluid [1-3]. Accumulation of succinylpurines is believed to exert neurotoxic effects and disrupt neuronal development and myelination, which may explain the severe hypotonia and early epileptic encephalopathy observed in neonatal ADSLD. The true clinical spectrum is wide, ranging from lethal fetal/neonatal encephalopathy to milder childhood forms with developmental delay and epilepsy; contemporary cohorts confirm marked heterogeneity and genotype-phenotype overlap [1,4,5].

The true incidence is unknown because diagnosis requires targeted succinylpurine analysis or molecular confirmation, and routine population-based newborn screening for ADSLD is not currently established [1,2,6,7]. A prevalence estimate based on aggregated exome data using Hardy-Weinberg assumptions is approximately one in 1.2-1.3 million individuals [6]. The number of published patients is increasing. A 2024 electroclinical appraisal identified 88 reported patients, among whom 12/88 (approximately 13.6%) had neonatal onset [5]. The most severe form may begin prenatally, with reported findings including reduced fetal movements or hypokinesia, growth restriction, and microcephaly, and postnatally it is characterized by profound hypotonia, early respiratory failure requiring ventilation, often refractory seizures, and early death [1,5,8,9].

## Case presentation

A female neonate was born at 34+0 weeks of gestation to a primigravida mother after a pregnancy complicated by mild polyhydramnios and progressive fetal microcephaly detected on prenatal ultrasound. Non-invasive prenatal testing was negative, and routine maternal screening for syphilis, human immunodeficiency virus (HIV), and hepatitis B was negative. Maternal oral glucose tolerance testing was normal. Abnormal Doppler findings were detected in the middle cerebral artery and ductus venosus, and cesarean delivery was therefore performed because of pathological Doppler flow patterns, suspicious cardiotocography (CTG), and suspected congenital developmental disorder (CDD) of the gastrointestinal tract. The Apgar scores were 3/3/8. A chronological overview of the subsequent clinical course and key diagnostic findings is summarized in Table 1.

**Table 1:** Timeline and key results. Abbreviations: GA = gestational age; DOL = day of life; CTG = cardiotocography; CDD = congenital developmental disorder; UApH = umbilical artery pH; BW = birth weight; NICU = neonatal intensive care unit; CXR = chest X-ray; VG = volume guarantee; PC = pressure control; FiO_2_ = fraction of inspired oxygen; MAP = mean airway pressure; nCPAP = nasal continuous positive airway pressure; DuoPAP = biphasic positive airway pressure; SIPPV = synchronized intermittent positive pressure ventilation; CHD = congenital heart disease; PFO = patent foramen ovale; PDA = patent ductus arteriosus; PN = parenteral nutrition; aEEG = amplitude-integrated electroencephalography; MRI = magnetic resonance imaging; IVH = intraventricular hemorrhage; ENT = ear, nose and throat; WGS = whole-genome sequencing; ADSLD = adenylosuccinate lyase deficiency; ADSL = adenylosuccinate lyase; CRP = C-reactive protein; I/T = immature-to-total neutrophil ratio; LP = lumbar puncture; CSF = cerebrospinal fluid. Note: The timeline separates neurological assessment, aEEG monitoring, infection evaluation, neuroinfection testing, hematology, urinary tract findings and supportive interventions into individual rows to improve readability.

Time (GA/DOL)	Event/Test	Key finding/Result	Intervention/Decision
34+0 weeks (birth)	Cesarean section for abnormal Dopplers/suspicious CTG, suspected CDD of the gastrointestinal tract	Apgar scores 3/3/8; no spontaneous respiration; marked hypotonia; UApH 7.33; lactate 3.5 mmol/L; BW 1660 g, approximately -1.1 SD by Fenton (2013) [10]	Ventilation; intubation at seven minutes of life; NICU admission
DOL1 (1 h 45 min)	Respiratory distress syndrome	'White lung' on chest X-ray; increasing oxygen requirement	Surfactant administration; invasive ventilation with volume guarantee
DOL1 and DOL3	Echocardiography	Hemodynamically stable without inotropic support; no major CHD; PFO and PDA; mild pulmonary hypertension	Conservative management
DOL1	Metabolic status	Hypoglycemia, 0.7 mmol/L	Glucose bolus; intravenous fluids
DOL1-DOL8	Feeding	Borderline feeding tolerance; continuous gastric tube feeding with mother's own milk and partial parenteral nutrition	Gradual escalation of enteral feeding
DOL1, DOL4 and DOL22	Serial cranial ultrasound	Evolving post-hemorrhagic hydrocephalus	Monitoring; later palliative approach
DOL2 and DOL16; daily bedside assessment throughout hospitalization	Neurological examination	Formal pediatric neurological examinations on DOL2 and DOL16; daily orientational neurological assessment by the attending physician. Findings included microcephaly, profound central hypotonia, weak/inconsistent suck reflex, absent Moro reflex, intermittent upper-limb hypertonia, stimulus-induced myoclonus, and jerky movements	Repeated neurological assessment; supportive care
DOL2-DOL4 and DOL14-DOL16	aEEG monitoring	Severely abnormal background with burst-suppression pattern; on DOL15, suspicious electroclinical activity was noted during marked motor restlessness, limb jerks, and myoclonus	aEEG monitoring; phenobarbital loading dose followed by maintenance therapy
DOL15 and DOL18	Clinically suspicious seizure activity/convulsions	DOL15: marked motor restlessness, jerky limb movements, and myoclonus with suspicious aEEG finding. DOL18: clinical jerks of both lower extremities; intermittent motor restlessness and myoclonus occurred during the course but were not always unequivocally epileptic	Phenobarbital started on DOL15; levetiracetam added on DOL18; subsequent clinical calming and reduction of sedative requirements
DOL2	TORCH evaluation	TORCH PCR and serology negative	Congenital infection not confirmed
Intermittently during hospitalization	Temperature instability	Intermittent temperature instability; non-specific finding evaluated in parallel with infectious work-up and clinical context	Symptomatic management; not interpreted as a diagnostic feature of ADSLD
DOL1-DOL3	Early sepsis screen/infection evaluation	DOL1: umbilical interleukin-6 negative; at seven hours of age, CRP 3.4 mg/L and I/T ratio 0.16. DOL2: Repeat tests for elevated temperature showed CRP 12 mg/L and I/T ratio 0.15; follow-up CRP 24 mg/L, with normothermia and stable clinical condition. DOL3: CRP increased to 33.5 mg/L with I/T ratio 0.4	Intravenous ampicillin plus gentamicin was started on DOL3 and continued for seven days
DOL3	Endocrinology	Suspected central hypothyroidism: TSH 3.750 mU/L, fT4 6.6 pmol/L	Levothyroxine started; dose titration according to follow-up tests
DOL4	First extubation attempt	Before extubation: pressure-control ventilation with volume guarantee 4 mL/kg, FiO_2_ 0.28, MAP 5 cmH_2_O, respiratory rate 58/min; the infant had spontaneous breathing. After extubation to nCPAP, MAP +7 cmH^2^O, FiO_2_ gradually increased to 0.50; after approximately one hour, escalation to DuoPAP up to 25 breaths/min. Increasing oxygen requirement and marked dyspnea developed despite apparently sufficient spontaneous effort; the clinical picture suggested upper-airway obstruction. Chest X-ray showed 'white lung.'	Reintubation after approximately two hours of non-invasive ventilation; SIPPV with volume guarantee started; oxygen requirement subsequently decreased
DOL8	Ophthalmology	No retinopathy of prematurity; markedly immature retina	Follow-up
DOL9	Feeding	Full enteral feeds achieved; slightly upward weight curve	Parenteral nutrition discontinued
DOL9	Brain MRI	Microcephaly; simplified gyrification; bilateral IVH; posterior fossa hemorrhage; delayed myelination	Prognostic counseling
DOL10	Second extubation attempt	Before extubation: pressure-control ventilation with volume guarantee 4.8 mL/kg, MAP 5 cmH_2_O, respiratory rate 58/min. Extubation to nCPAP/DuoPAP was again followed by marked dyspnea and restlessness. Fresh blood from the airways was noted during reintubation	Reintubation after approximately 20 minutes; continued invasive ventilation
DOL10 and DOL21-DOL23	Genetics and metabolic testing	Normal karyotype. Rapid WGS through the Baby Fox program confirmed autosomal recessive ADSLD. Metabolic testing demonstrated ADSL deficiency consistent with the most severe neonatal form; no causal treatment is available.	Genetic and metabolic counseling; reassessment of goals of care
DOL14	ENT consultation	No structural upper-airway abnormality identified	No surgical intervention indicated
DOL14	Bronchoscopy	Right upper-lobe tracheal bronchus, interpreted as an anatomical variant	No causal airway lesion explaining the full clinical course
DOL14	Hematology	Anemia: Hb 91 g/L, Hct 27%	Packed red blood cell transfusion
From DOL1, intermittently	Icterus	Recurrent hyperbilirubinemia without incompatibility	Intermittent phototherapy
From DOL1, intermittently	Microbiology/colonization surveillance	Surveillance cultures intermittently positive for colonization, including Escherichia coli; no congenital infection confirmed	Antibiotic treatment guided by clinical context and inflammatory markers
DOL19-DOL29	Urinary tract	Urinary retention	Permanent urinary catheterization
DOL21	Sepsis work-up	Morning CRP 30 mg/L, evening CRP 192 mg/L; lactate 3.5 mmol/L; I/T ratio 0.35. Clinically, without major deterioration, hemodynamically stable, afebrile, central venous catheter site calm; FiO_2_ increased from approximately 0.23 to 0.30	Piperacillin/tazobactam plus vancomycin
DOL21	Neuroinfection evaluation, LP/CSF	Lumbar puncture performed; CSF testing showed no evidence of neuroinfection; PCR/cultures negative	Continued antibiotic therapy according to clinical and laboratory course
DOL29	Outcome	Death	Comfort-focused palliative care

After birth, the neonate had no spontaneous respiratory effort and marked hypotonia, requiring ventilation and intubation at seven minutes of life. Umbilical artery pH was 7.33, and lactate was 3.5 mmol/L. Birth weight was 1660 g (approximately -1.1 SD), and head circumference corresponded to -2.6 SD by Fenton (2013) [10]. Shortly after, severe pneumopathy/respiratory distress syndrome (RDS) developed, which prompted surfactant administration at one hour and 45 minutes of life. Oxygenation subsequently improved, and mechanical ventilation using pressure-control ventilation with volume guarantee was continued. Ventilatory requirements initially remained low (fraction of inspired oxygen (FiO₂) 0.21-0.35). The first extubation attempt was performed on day of life (DOL) 4. Before extubation, the infant was ventilated in pressure-control mode with volume guarantee (VG 4 mL/kg), FiO₂ 0.28, mean airway pressure (MAP) 5 cmH₂O, and a ventilator rate of 58/min, with some spontaneous respiratory effort. Extubation was initially to non-invasive continuous positive airway pressure (nCPAP) with MAP 7 cmH₂O; FiO₂ had to be increased gradually to 0.50. After approximately one hour, support was escalated to DuoPAP with rates up to 25/min because of increasing oxygen requirements and marked dyspnea. Although spontaneous respiratory effort appeared sufficient, the clinical picture resembled upper-airway obstruction at the tongue/throat level. A chest X-ray showed a “white lung” appearance, and the infant was reintubated after approximately two hours of non-invasive ventilation and placed on synchronized intermittent positive-pressure ventilation (SIPPV) with VG, after which oxygen requirements could be reduced. The second extubation attempt was performed on DOL10. Before extubation, ventilator settings were pressure-control ventilation with VG 4.8 mL/kg, MAP 5 cmH₂O, and a ventilator rate of 58/min. After extubation to nCPAP/DuoPAP, a similar pattern of marked dyspnea and agitation occurred, and reintubation was required after approximately 20 minutes. Fresh blood was observed from the airway during intubation. Ear, nose, and throat consultation did not reveal a fixed obstructive lesion. Bronchoscopy showed a tracheal bronchus supplying the upper lobe of the right lung, interpreted as an anatomical variant rather than the primary reason for extubation failure. Hemodynamics remained stable without inotropic support.

Neurological examination was performed immediately after birth and then daily in orienting form by the attending physician; formal pediatric neurological examinations were performed on DOL2 and DOL16. The examination showed markedly decreased muscle tone from birth, with intermittent hypertonia in the upper extremities. Craniofacial examination showed mild dysmorphic features and a small anterior fontanelle. Primitive reflexes were abnormal: the suck reflex was weak and inconsistent, and the Moro reflex was absent. Repetitive stimulus-induced jerks, intermittent motor unrest, myoclonus, and jerky limb movements were observed, although these early motor phenomena were not unequivocally interpreted as seizures. Amplitude-integrated electroencephalography (aEEG) demonstrated a burst-suppression background pattern suggestive of severe epileptic encephalopathy. On DOL15, aEEG was restarted because of motor agitation. After approximately 10 hours of marked motor agitation, jerky limb movements, and myoclonus with suspicious aEEG findings, phenobarbital was administered as loading and maintenance therapy, after which the motor agitation decreased. On DOL18, levetiracetam was added for clinical twitching of both lower extremities; at that time, aEEG had already been discontinued for two days. Thereafter, the infant became calmer, and sufentanil dosing could be reduced. Intermittent temperature instability was observed during the clinical course. Because infectious evaluation was repeatedly assessed in parallel and because the infant had severe structural and functional central nervous system involvement, central thermoregulatory dysregulation was considered as a possible contributing factor; however, this finding was non-specific and was not interpreted as a diagnostic feature of ADSLD.

Brain MRI performed on DOL9 showed microcephaly with simplified cortical gyrification, bilateral intraventricular hemorrhage, posterior fossa hemorrhagic changes, and delayed myelination, supporting the presence of severe structural brain involvement in addition to the abnormal neurological examination and aEEG background (Figure 1).

**Figure 1:**
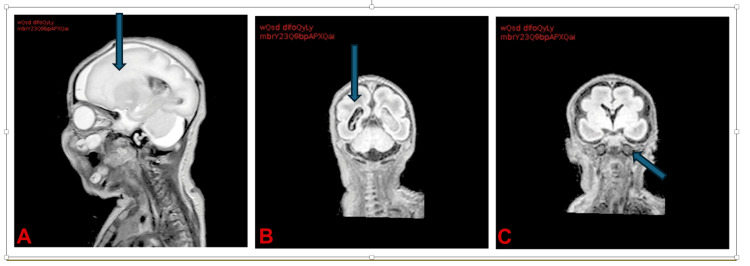
MRI of the brain (A) Sagittal brain MRI demonstrating microcephaly and simplified cortical gyrification. (B) Coronal brain MRI showing intraventricular hemorrhage and structural brain immaturity. (C) Coronal brain MRI demonstrating hemorrhagic changes within the posterior fossa.

Small bilateral periventricular hypoxic foci were considered most likely related to IVH. White matter myelination was delayed for corrected age (e.g., no myelination of the posterior limb of the internal capsule), raising concern for hypomyelination or another myelin disorder. 

Infectious and inflammatory screening was performed from the first DOL. Blood tests immediately after delivery showed negative umbilical interleukin-6 (IL-6). At seven hours of age, C-reactive protein (CRP) was 3.4 mg/L, and the immature-to-total neutrophil (I/T) ratio was 0.16. On DOL2, because of elevated temperature, repeat testing showed CRP 12 mg/L and I/T ratio 0.15; a subsequent control showed CRP 24 mg/L while the infant was normothermic and clinically stable. On DOL3, CRP increased to 33.5 mg/L with an I/T ratio of 0.40, and ampicillin plus gentamicin was started despite no major clinical deterioration. A later sepsis evaluation was performed on DOL21 after daily CRP values had been only mildly elevated (approximately 15 mg/L) and then increased to 30 mg/L in the morning and 192 mg/L in the evening, with lactate at 3.5 mmol/L and I/T ratio 0.35. Lumbar puncture showed no evidence of neuroinfection. Vancomycin plus piperacillin/tazobactam was initiated. At that time, the infant was clinically without major new findings, hemodynamically stable, afebrile, and the central venous catheter site was calm; the main concurrent change was an increase in FiO₂ requirement from approximately 0.23 to 0.30.

Given the persistent severe neurological phenotype, abnormal aEEG background, and subsequent MRI abnormalities, targeted metabolic testing and genetic testing were initiated in parallel during the diagnostic work-up. Urine and serum samples for succinylpurine analysis were sent to the Diagnostic and Research Unit for Rare Diseases at the 1^st^ Faculty of Medicine, Charles University, and General University Hospital in Prague, where they were analyzed using high-performance liquid chromatography. Following a normal karyotype result, rapid trio WGS of the patient and both parents was initiated within the national Baby Fox program at University Hospital Brno. Thus, biochemical testing was requested after early severe encephalopathy had become clinically evident and before the final molecular diagnosis; metabolic and WGS results became available within the same diagnostic interval, allowing biochemical-molecular correlation before the transition to palliative care.

The metabolic analysis demonstrated markedly elevated levels of urinary and serum S-Ado and SAICAr. These findings are summarized in Table 2.

**Table 2:** Metabolic analysis of succinylpurines in urine and serum These results demonstrate marked accumulation of succinylpurines in both urine and serum, representing the biochemical hallmark of adenylosuccinate lyase deficiency. The markedly elevated urinary concentrations, together with detectable serum levels that are normally below the limit of quantification, strongly support the diagnosis in the clinical context.

Parameter	Sample	Value	Reference range
Succinyladenosine (S-Ado)	Urine	192 mmol/mol creatinine	≤7 mmol/mol creatinine
SAICA-riboside (SAICAr)	Urine	305 mmol/mol creatinine	Not detected, below the limit of quantification
S-Ado	Serum	23.3 µmol/L	Not detected, below the limit of quantification
SAICAr	Serum	1.4 µmol/L	Not detected, below the limit of quantification

On the same day that the metabolic results became available, rapid WGS reported two variants in the ADSL gene occurring in trans: a maternally inherited likely pathogenic variant NM_000026.4:c.1343_1345del (p.Ser448del) and a paternally inherited variant of uncertain significance (VUS) NM_000026.4:c.1106T>C (p.Ile369Thr). In conjunction with the characteristic biochemical profile and clinical presentation, these findings established the diagnosis of ADSLD.

The paternally inherited NM_000026.4:c.1106T>C (p.Ile369Thr) variant was classified as a variant of uncertain significance (VUS) and was, therefore, not considered diagnostic on its own. Its possible contributory role was interpreted cautiously in the context of confirmed trans inheritance with the maternally inherited likely pathogenic NM_000026.4:c.1343_1345del (p.Ser448del) variant, the highly characteristic biochemical profile showing marked accumulation of succinylpurines, and the severe neonatal phenotype compatible with ADSLD. Thus, the diagnosis was supported primarily by the concordance between the clinical presentation, biochemical signature, and biallelic ADSL variants, while the uncertain classification of p.Ile369Thr was retained.

## Discussion

This case illustrates the most severe neonatal phenotype of ADSLD, characterized by probable prenatal onset, marked microcephaly, profound central hypotonia, and early epileptic encephalopathy. Although the phenotypic spectrum of ADSLD is broad, neonatal onset appears to represent only a minority of all reported patients (12/88 in a 2024 appraisal) and is associated with particularly poor prognosis and early mortality [5].

In our patient, metabolic and genetic testing were initiated in parallel. Detection of elevated succinylpurines (S-Ado, SAICAr) provided the characteristic biochemical signature of ADSLD, while rapid trio WGS established the molecular diagnosis within a clinically relevant timeframe. Rather than implying a single universal diagnostic standard, the present case supports the complementary value of biochemical succinylpurine detection and molecular confirmation in suspected ADSLD [1,2,7]. In neonatal intensive care unit (NICU) practice, rapid whole-exome sequencing (WES) or WGS is increasingly used in selected critically ill neonates with suspected monogenic disease; however, availability, turnaround time, interpretation expertise, and clinical applicability vary across healthcare systems [11-14]. In appropriate settings, rapid genomic testing may shorten the diagnostic odyssey, facilitate prognostic counseling, inform decisions regarding invasive interventions, and support shared decision-making with families.

Neuroimaging findings in our patient should be interpreted cautiously because prematurity can contribute to several abnormalities. Bilateral intraventricular hemorrhage (IVH), evolving post-hemorrhagic hydrocephalus, and small periventricular hypoxic foci may be partly or substantially related to prematurity and neonatal illness. In contrast, antenatal progressive microcephaly, simplified gyrification, delayed myelination for corrected age, profound hypotonia from birth, and a burst-suppression aEEG background were less readily explained by prematurity alone and supported an underlying primary severe central nervous system disorder in the overall clinical context. Accumulation of succinylpurines may interfere with neuronal maturation and myelination and may also reflect broader cellular dysfunction, including mitochondrial and signaling pathway disturbances [3,15].

Beyond the immediate diagnostic implications, establishing the molecular diagnosis has important consequences for family counseling. In families with known pathogenic ADSL variants, targeted prenatal diagnosis from fetal DNA using chorionic villus sampling (CVS) or amniocentesis can be offered in future pregnancies, and reproductive options such as preimplantation genetic testing for monogenic disease (PGT-M) may be discussed [16,17].

Prenatal manifestations, including microcephaly and abnormal fetal findings, are uncommon in the published ADSLD literature but have been described in the most severe fetal and early neonatal presentations [1,8,9]. Because the evidence is based on small case series and individual reports, this association should be interpreted cautiously. Available reports suggest, but do not prove, that prenatal manifestations may mark a particularly severe disease trajectory. Our case adds to this limited literature by documenting progressive prenatal microcephaly, abnormal fetal Doppler findings, severe neonatal encephalopathy, and rapid clinical deterioration. A neonatal case diagnosed by whole-exome sequencing has also been reported [18]. The integration of biochemical testing and rapid WGS allowed confirmation within a clinically actionable timeframe. This supports considering rapid WES/WGS, in parallel with targeted metabolic analysis, in selected critically ill neonates with severe unexplained encephalopathy, especially when conventional diagnostics are inconclusive.

Limitations of this case report include the absence of a full conventional EEG (monitoring relied on aEEG) and the lack of precision in certain timeline items (DOL), which should be harmonized with the full medical record.

In conclusion, this case highlights the importance of considering rare monogenic metabolic disorders such as ADSLD in neonates presenting with severe encephalopathy, microcephaly, and profound hypotonia from birth. Early implementation of rapid genomic testing, when available and appropriate, in parallel with targeted metabolic analysis, enabled etiologic diagnosis within a clinically meaningful timeframe. In critically ill neonates with unexplained neurological deterioration, such an approach may shorten the diagnostic process and provide timely information for prognostic assessment and further decision-making. However, feasibility and impact may vary by setting.

Take-home message

In neonates with microcephaly, profound hypotonia, and early epileptic encephalopathy, ultra-rare disorders of purine metabolism such as ADSLD should be considered [1,3]. Prenatal findings such as progressive microcephaly, polyhydramnios, or abnormal Dopplers may be an early signal of a severe phenotype. Still, this association is based on limited published evidence and should be interpreted cautiously [1,8,9]. In selected NICU settings, rapid WGS/WES may accelerate etiologic diagnosis, improve prognostication, and support shared decision-making on goals of care; however, access, turnaround time, and clinical utility vary across healthcare systems [11-14]. After diagnosis, early involvement of a metabolic specialist, neurologist, and palliative care team is essential; no disease-modifying therapy is currently available [1].

## Conclusions

ADSLD should be considered in neonates presenting with severe microcephaly, profound hypotonia, and early epileptic encephalopathy. Timely molecular diagnosis, ideally through rapid genomic sequencing where available and clinically appropriate, can provide diagnostic certainty within a clinically meaningful timeframe and support prognostic assessment and ethical decision-making. For suspected ADSLD, molecular findings should be interpreted together with the biochemical succinylpurine profile and the clinical phenotype.
